# Testing the Direction of Longitudinal Paths between Victimization, Peer Rejection, and Different Types of Internalizing Problems in Adolescence

**DOI:** 10.1007/s10802-016-0216-y

**Published:** 2016-10-26

**Authors:** Miranda Sentse, Peter Prinzie, Christina Salmivalli

**Affiliations:** 1 Institute for Criminal Law and Criminology, Leiden University, P. O box 9520, 2300 RA Leiden, the Netherlands; 2 Department of Psychology, Education & Child Studies, Erasmus University Rotterdam, Rotterdam, the Netherlands; 3 Department of Psychology, University of Turku, Turku, Finland

**Keywords:** Depression, Anxiety, Victimization, Social status, Adolescence

## Abstract

The transition to secondary school is accompanied by the fragmentation of peer groups, while adolescents are also confronted with heightened incidents of bullying and increased levels of internalizing problems. Victimization, peer rejection, and internalizing problems are known to be interrelated, but how they influence each other over time remains unclear. We tested the direction of these associations by applying a cross-lagged path model among a large sample of Finnish adolescents (*N* = 5645; 49.1 % boys; *M* age at T1 = 14.0 years) after they transitioned to secondary school (grades 7–9). Self-reported depression, anxiety, and victimization and peer-reported rejection were measured 3 times over the course of 1 year. Results showed that depression was predictive of subsequent victimization for both boys and girls, in line with a symptoms-driven model; for girls, anxiety was reciprocally related to victimization, in line with a transactional model; for boys, victimization was related to subsequent anxiety, in line with an interpersonal risk model. Peer rejection was not directly related to depression or anxiety, but among girls peer rejection was bi-directionally related to victimization. Overall, our results suggest that associations between internalizing problems and peer relations differ between depression and anxiety and between genders. Implications for practice and directions for future research are discussed.

Adolescence is a period characterized by major changes in multiple domains including biological, cognitive, relational, and behavioral changes (Eccles et al. 1993). The transition to secondary school in particular brings about several changes, mainly because secondary schools are larger with more diverse peer groups than primary schools. While peer relations become increasingly important, adolescents’ peer groups are shaken up and their social status in the classroom needs to be (re-)established when entering the new, larger school. This social restructuring is claimed to cause a rise in aggression and more specifically in bullying, as bullying can be used as a means to acquire or maintain popularity (Pellegrini and Long 2002). The likelihood of (temporary) increased incidents of bullying is further supported by the finding that adolescents place more importance on popularity than on socially accepted behaviors (LaFontana and Cillessen 2010). Transitioning to secondary school and social restructuring are not only related to increases in bullying and victimization, but also to increases in internalizing problems, such as feelings of loneliness, depressed mood, and social anxiety (Eslea et al. 2004; Hankin and Abramson 2001). The transition may partly explain why most depressive and anxiety symptoms have their onset in early adolescence, and increase and peak in adolescence (Hankin and Abramson 2001; Twenge and Nolen-Hoeksema 2002).

Taken together, internalizing problems and negative peer relations including peer rejection and victimization are likely interrelated, especially during adolescence. But although associations between peer victimization and internalizing problems have been well documented (see Reijntjes et al. 2010), the direction of effects and the role of peer rejection therein remain rather unclear, particularly in adolescence. Given the importance of the transition to adolescence, the current study aimed to test the direction of effects by applying a model with cross-lagged paths between victimization, peer rejection, and internalizing problems among adolescents after they transitioned to secondary school (grades 7–9 corresponding to age 13–16 years). In addition, because studies have shown that etiological factors of anxiety and depression differ (Prinzie et al. 2014), we investigated separate models for depressive and anxiety symptoms and tested for possible gender differences in all associations.

## Theoretical Framework

The association between negative peer relations and internalizing problems in adolescence can be studied from three different theoretical perspectives (cf. Kochel et al. 2012); a symptoms-driven model in which internalizing problems predict negative peer relations, an interpersonal risk model in which negative peer relations predict internalizing problems, and a transactional model in which negative peer relations and internalizing problems influence each other over time. The three models including associated findings from previous research will discussed below.

First, a *symptoms-driven model* states that depressed or anxious individuals show a distinctive pattern of social behavior which elicits a negative response from others. For example, it was found that interactions with persons who are depressed induce a more depressive mood than interactions with non-depressed persons (Hammen and Peters 1978). Research on the peer networks among depressed adolescents have found that depressed adolescents are more often left alone and have a more problematic status in the peer group (Kaltiala-Heino et al. 2010; Rudolph and Clark 2001). Moreover, depressive affect has been found to predict peer rejection a few months later (Vernberg 1990). Similarly, it has been suggested that the withdrawn behavior of anxious or depressed children makes them less likely to defend themselves and increases the chances to be singled out and attacked by bullies (Hodges & Hodges and Perry 1999). Indeed, several studies have found internalizing problems to predict subsequent peer rejection and victimization (Brock et al. 2006; Paul and Cillessen 2003).

Interestingly, studies that differentiated between depression versus anxiety, and/or boys and girls, report inconsistent findings. For example, Kochel et al. (2012) found internalizing problems predictive for victimization and lower peer acceptance in late childhood (grades 4–6), in line with findings of Kaltiala-Heino et al. (2010) who studied adolescents, but both only focused on depression. Similarly, Lester et al. (2012) found depression predictive of victimization but this effect only held for adolescent girls, whereas in another study this effect only held for adolescent boys (Sweeting et al. 2006). In contrast, other studies have reported no gender differences in associations between internalizing problems and victimization (Tran et al. 2012; Vaillancourt et al. 2013) or peer rejection (Agoston and Rudolph 2013; Kaltiala-Heino et al. 2010).

Second, contrary to a symptoms-driven model, the *interpersonal risk model* highlights the role of social relationships in the development and maintenance of psychopathology. For example, according to interpersonal models depression and anxiety are not simply a consequence of cognitive distortion, but arise in a certain social environment especially when relationships are conflicting and unsupportive (Hammen 1992). That is, being bullied, peer rejection, or a lack of friendships is likely to lead to feelings of loneliness and interferes with the basic human need to belong (Baumeister and Leary 1995) which can ultimately lead to more severe internalizing problems such as depression and (social) anxiety. In line with this model, it has been found that victimization is associated with subsequent internalizing problems in young children (Arseneault et al. 2006) and late childhood (Williford et al. 2012), and this effect even lasts into late adolescence and adulthood (for reviews see McDougall and Vaillancourt 2015; Ttofi et al. 2011). Yet some studies only found such an effect for girls but not for boys (e.g., Bond et al. 2001).

Lastly, *a transactional model* acknowledges the dynamic interplay between an individual and the (social) context (Sameroff and MacKenzie 2003) and combines the previous two models by suggesting that internalizing problems may cause individuals to trigger negative peer reactions (e.g., peer rejection, victimization) which in turn can contribute to even more internalizing problems and vice versa. Victimized youth are often described as unpopular and tend to be highly disliked (i.e., rejected) by peers (Prinstein and Cillessen 2003) and adolescents with vulnerabilities such as internalizing problems seem to be victimized more when they are not socially protected by peers (Hodges et al. 1997). Thus, adolescents who are socially rejected may be easy targets for bullies. Bullies are less likely to be confronted by defenders of victims and rejected adolescents might spend more time being alone, which again increases the risk of internalizing problems. Also, victims may be rejected because having a friendship with a victim may decrease someone’s own social status in the peer group (Sentse et al. 2013).

In line with a transactional model, a meta-analysis on longitudinal studies found evidence for both pathways, that is, internalizing problems predicted victimization over time and vice versa (Reijntjes et al. 2010). However, to test or conclude about a transactional model, studies that include both pathways simultaneously are a prerequisite. These studies, however, are scarce and the few available studies report inconsistent findings. Lester et al. (2012) found transactional paths between anxiety and victimization for both genders, and between depression and victimization for adolescent males only. Other studies analyzed transactional models but, as described earlier, found only evidence for a one-way path between internalizing problems and victimization (Sweeting et al. 2006; Tran et al. 2012; Vaillancourt et al. 2013) and/or peer rejection (Agoston and Rudolph 2013; Kochel et al. 2012).

In sum, empirical evidence for each of the three theoretical models exists, however, it is the inconsistency in research findings together with methodological shortcomings of some studies that makes it challenging to draw any firm conclusions. More specifically, all three theoretical models call for longitudinal designs and to conclude on any specific model it is necessary that both directions of all associations between peer rejection, victimization, and depression and anxiety are examined simultaneously and longitudinally in a cross-lagged framework, yet so far this has not been done (see for an exception Kochel et al. 2012, who examined children in grades 4–6).

## The Current Study

To contribute to the extant literature and to overcome methodological shortcomings some studies were faced with (that is, the lack of a cross-lagged framework), the current study is one of the first to test one model that included concurrent as well as cross-lagged associations between victimization, peer rejection, and internalizing problems. Moreover, the model was tested in a large sample of adolescents after they had transitioned to secondary school (grades 7–9, aged 13–16 years) over the course of 1 year. Because studies have shown that etiological factors of anxiety and depression differ (Prinzie et al. 2014), and that associations with victimization and peer status differ between the two types of internalizing problems, we tested the models separately for anxiety and depression. However, due to the inconsistent findings from previous research no specific hypotheses could be made for differences between depression and anxiety models.

Additionally, we tested for gender differences as it is likely that associations between victimization, peer rejection, and internalizing problems differ between boys and girls, especially regarding depression. First, prevalence rates of depression are higher for girls than for boys (Hankin and Abramson 2001) and more girls than boys increase in internalizing symptoms in the adolescent years (e.g., Angold et al. 2002). In addition, it is known that low levels of peer support (e.g., peer rejection) have a stronger effect on girls than on boys and that conversely, among adolescent girls depressive symptoms predict a decline in peer support (Stice et al. 2004). In coping with depression, however, adolescent girls are more likely to intensify their depressed mood through rumination with friends about their mood. Co-rumination refers to an extensive focus on negative feelings, and discussing and speculating about problems (Rose 2002). In contrast, boys rather distract themselves from negative cognitions, which may dampen their depressed mood (Nolen-Hoeksema 1991). Thus, girls may be more vulnerable for negative peer relations and are more likely to participate in social activities that intensify their depressed mood. Therefore, for girls we hypothesized to find stronger associations between peer victimization, peer rejection, and depression, irrespective of the direction of these associations. Gender differences in the model including associations with anxiety were also tested but based on available literature no clear hypotheses could be made.

## Method

### Participants and Procedure

Data came from the KiVa antibullying program evaluation in grades 7 to 9 (see Kärnä et al. 2013 for details on the intervention program). In Finland, grade 7 to 9 (i.e., age 13–16) may best be described as lower secondary school, as the term upper secondary school is used there to describe grade 10–12 (i.e., age 16–18). For the sake of clarity, we consistently use the term secondary school in this study. In the fall of 2006, recruitment letters were sent to all 3418 schools in mainland Finland. The 275 volunteering schools were stratified by province and language and after exclusion of special-education-only schools, 78 secondary schools with grades 7–9 representing all five provinces in mainland Finland were randomly assigned to the intervention or control condition (39 schools each). Subsequently, parents were sent information letters including an active consent form. Active parental consent was obtained from 87.4 % of the target sample. Four control schools dropped out without providing any data, and one intervention school participated only in the first wave of data collection. After excluding these five schools, we were left with 38 intervention schools and 35 control schools. For the current study we used data from control schools only to avoid unrepresentative associations between the study variables due to the intervention.

Data were collected over the course of 1 year in May 2008 (T1; grades 7–8), December 2008 (T2; grades 8–9), and May 2009 (T3; grades 8–9). Our sample consisted of 5645 students (49.1 % boys; T1 *M* age = 14.0 years, *SD* = 0.83), excluding 88 students with missing values on all study variables. Most students were native Finns, the proportion of immigrants being 2.5 %. Of the 5645 students, 53.9 % participated in all three waves. Attrition analysis revealed that of all measures used in the current study, significant mean differences existed between three-wave responders and those for whom at least one data wave was missing in victimization at T1, *t*(2084) = 1.99, *p* < 0.05 and T2, *t*(2405) = 1.99, *p* < 0.05 and in peer rejection at T1, *t*(4098) = 2.98, *p* < 0.01, and T3, *t*(3590) = 4.21, *p* < 0.01, with three-wave responders having lower mean scores on victimization and peer rejection than the others (effect sizes ranged between *d =* 0.07 and *d =* 0.13). For the current study, all available pieces of information of the 5645 students were used (see analytical strategy).

Students completed internet-based questionnaires during regular school hours, under supervision of their teachers who received detailed instructions two weeks prior to data collection. The teachers were told to act in such ways that the confidentiality of the response was secured to a maximum extent. In addition, teachers were offered support through phone or e-mail prior to and during data collection. The sessions took on average 21 minutes. Students were assured that their answers would not be revealed to teachers or parents and that their participation was voluntary. The order of the questions, items, and scales in the questionnaire was extensively randomized to alleviate any systematic order effect. All the measures (see below) were translated and back-translated either by a professional translator or a native English-speaker. The victimization measure had already an existing Finnish version.

### Measures

#### Victimization

At the beginning of the questionnaire, the term “bullying” was defined for the students based on the Olweus’ (1996) definition, which emphasizes the repetitive nature of bullying and the power imbalance between bully and victim. Victimization was measured with the revised Olweus Bully ⁄ Victim Questionnaire (Olweus 1996). One general question, “How often have you been bullied at school in the last couple of months?” was followed by 10 questions tapping into specific forms of victimization (e.g., “I was hit, kicked, or pushed”, “I was called nasty names or laughed in my face or hurt by insults”). Students answered all questions on a 5-point scale (0 = *not at all*, 4 = *several times a week*). The scores on the 11 items were averaged (Cronbach’s alphas ranged from 0.91 to 0.94 across waves).

#### Peer Rejection

Students were asked to nominate the classmates they liked least to assess peer rejection (cf. Coie et al. 1982). Students could nominate an unlimited number of peers. The class roster consisted of all the students in the classroom, but peer nominations for adolescents without parental consent were not further used in the study. To account for differences in classroom size and thus possible nominators, for each student the received nominations were summed and divided by the number of nominators. As such, scores on peer rejection could vary from 0.00 to 1.00 (proportion scores). In order to make sure that the interpretation of the peer nominations was valid in all classrooms, we checked the percentage of adolescents with parental consent to participate per classroom. All classrooms except for 4 % at T1, had percentages of parental consent above 75 % with a mean percentage of 86 %.

#### Depression

Students’ level of depression was measured by a 7-item scale derived from the Beck Depression Inventory (BDI; Beck et al. 1996). As the BDI was previously translated into Finnish and validated in two prior studies (Raitasalo 1977, 2007), we chose this measure over the CDI. Items were selected based on their suitability for use with children and early adolescents. Items regarding suicidal ideation and intent, sexual interest, and somatic complaints (e.g., losing appetite, losing weight, and being worried about one’s health) were eliminated, resulting in a 7-item scale that assessed cognitive-affective concerns. The scale consists of statements such as “What is your mood like?” and “How do you feel about yourself?” which were rated on a 5-point scale (0 = *fairly bright and good*, 4 = *completely unhappy*), evaluating the past two weeks. Scores on the seven items were averaged to create a depression scale. Cronbach’s alphas were good across all three waves (α = 0.89–0.94). Because we look at depressive symptoms as a continuous variable and do not categorize students into clinically depressed versus non-depressed, the shortened version should be highly correlated with the original total scale score.

#### Anxiety

Two social anxiety scales, the Fear of Negative Evaluation and the Social Avoidance and Distress, were combined to measure students’ level of anxiety (García-López et al. 2001). Five items tapped into the extent to which others’ evaluation of the student causes undue stress and worry (e.g., “I’m afraid the others won’t like me”) and four items tapped into the extent to which students avoid social interactions and feel uncomfortable in group situations (e.g., “I stay quiet when I’m in a group of people). Students rated each statement on a 5-point scale ranging from 0 (*not at all*) to 4 (*all the time*). As we had no reason to expect that the two aspects of social anxiety would be differently related to victimization, the nine items were averaged to compute anxiety scores (Cronbach’s alphas ranged from 0.92 to 0.94 across waves).

### Statistical Analyses

Cross-lagged path models were tested in Mplus 7.11 (Muthén and Muthén 1998-2012) using full information maximum likelihood estimation with robust standard errors (MLR). When using MLR, missing values are not replaced or imputed but the missing data is handled within the analysis model. This estimation procedure is preferred over conventional linear regression, because MLR takes into account all available pieces of information, avoids listwise or pairwise deletion, and corrects for multivariate non-normality in the data. By using the “cluster” option in Mplus, analyses were additionally corrected for dependencies in our data due to student clustering at the classroom level.

Two cross-lagged path models were computed (i.e., depression-rejection-victimization and anxiety-rejection-victimization), including stability of the variables and concurrent associations between the variables. Moderation by gender was tested via multi-group analyses. We constrained paths in each model to be equal for both genders and compared this constrained model to an unconstrained model in which paths were free to vary across gender. Model fit was compared with the Satorra-Bentler difference test (Satorra and Bentler 2001) which is used in a similar fashion as a standard *χ* ^*2*^ difference test but accounts for MLR estimation. If a fully constrained model fits the data equally well as an unconstrained model, the constrained model is favored in terms of model parsimony. The model fit of each final model was evaluated with the Comparative Fit Index (CFI), the Root Mean Error of Approximation (RMSEA), and the Standardized Root Mean Square Residual (SRMR). Acceptable model fit is indicated by values of 0.95 or higher for the CFI, lower than .06 for the RMSEA, and lower than .08 for the SRMR (Hu and Bentler 1999).

## Results

### Descriptive Statistics

Table 1 shows the means of all study variables for the total sample as well as for boys and girls separately. Gender differences were present in all study variables. Across all three waves, boys scored higher than girls on victimization and peer rejection, whereas girls scored higher than boys on depression and anxiety (effect sizes ranged between *d =* 0.07 and *d =* 0.28). Correlations between the study variables are reported in Table 2. For both genders, all measures were relatively stable over time (for most variables *r* ranged between 0.35 and 0.70 across time points). Across time and gender, victimization, peer rejection, depression, and anxiety were all positively correlated with each other, except for the non-significant associations between peer rejection (across all waves) and anxiety at T3 for boys.

**Table 1:** Means and standard deviations of the study variables for the total sample and separately by gender

	Total		Girls		Boys		Difference		Effect size
Variable	Mean	SD	Mean	SD	Mean	SD	*t*	*df*	Cohen’s d
Victimization T1	0.24	0.44	0.20	0.33	0.29	0.53	-7.07**	4366	0.21
Victimization T2	0.21	0.46	0.16	0.33	0.26	0.56	-7.51**	4475	0.22
Victimization T3	0.22	0.55	0.16	0.38	0.29	0.68	-7.26**	3939	0.23
Peer rejection T1	0.15	0.16	0.14	0.15	0.17	0.17	-6.88**	5135	0.19
Peer rejection T2	0.15	0.15	0.14	0.14	0.16	0.16	-6.08**	5360	0.17
Peer rejection T3	0.13	0.15	0.13	0.15	0.14	0.15	-2.37*	4984	0.07
Depression T1	0.77	0.75	0.85	0.75	0.69	0.73	7.00**	4295	0.21
Depression T2	0.80	0.78	0.89	0.79	0.70	0.76	8.10**	4430	0.24
Depression T3	0.79	0.90	0.86	0.86	0.72	0.93	5.00**	3796	0.16
Anxiety T1	1.32	0.78	1.41	0.73	1.22	0.82	8.00**	4291	0.24
Anxiety T2	1.29	0.79	1.40	0.74	1.18	0.83	9.22**	4422	0.28
Anxiety T3	1.31	0.86	1.41	0.79	1.20	0.92	7.57**	3793	0.24

*T1* = Time 1, *T2* = Time 2, *T3* = Time 3. ** = *p* < 0.01; * = *p* < 0.05

**Table 2:** Correlations between the study variables, for girls below the diagonal and for boys above the diagonal

	1	2	3	4	5	6	7	8	9	10	11	12
1. Victimization T1	-	0.42	0.27	0.26	0.22	0.20	0.42	0.23	0.17	0.25	0.18	0.11
2. Victimization T2	0.50	-	0.42	0.20	0.22	0.18	0.24	0.34	0.25	0.12	0.17	0.07
3. Victimization T3	0.36	0.41	-	0.11	0.13	0.14	0.22	0.25	0.45	0.06	0.09	0.09
4. Peer rejection T1	0.30	0.25	0.19	-	0.67	0.61	0.14	0.10	0.07	0.07	0.08	**0.01**
5. Peer rejection T2	0.25	0.25	0.18	.65	-	0.68	0.13	0.12	0.10	0.06	0.07	**0.01**
6. Peer rejection T3	0.23	0.23	0.19	0.55	0.66	-		0.10	0.10	**0.04**	0.05	**0.00**
7. Depression T1	0.33	0.22	0.20	0.14	0.10	0.11	-	0.47	0.36	0.21	0.14	0.10
8. Depression T2	0.22	0.31	0.25	0.12	0.11	0.11	0.62	-	0.49	0.19	0.23	0.12
9. Depression T3	0.22	0.21	0.34	0.11	0.09	0.11	0.57	0.70	-	0.09	0.12	0.13
10. Anxiety T1	0.26	0.18	0.10	0.13	0.12	0.14	0.40	0.30	0.30	-	0.38	0.24
11. Anxiety T2	0.20	0.18	0.14	0.10	0.11	0.09	0.36	0.42	0.31	0.55	-	0.33
12. Anxiety T3	0.19	0.22	0.15	0.08	0.08	0.09	0.27	0.32	0.38	0.48	0.55	-

*T1* = Time 1, *T2* = Time 2, *T3* = Time 3. All correlations are significant at *p* < 0.05 except for correlations in bold

### Cross-Lagged Path Models

**Fig. 1:**
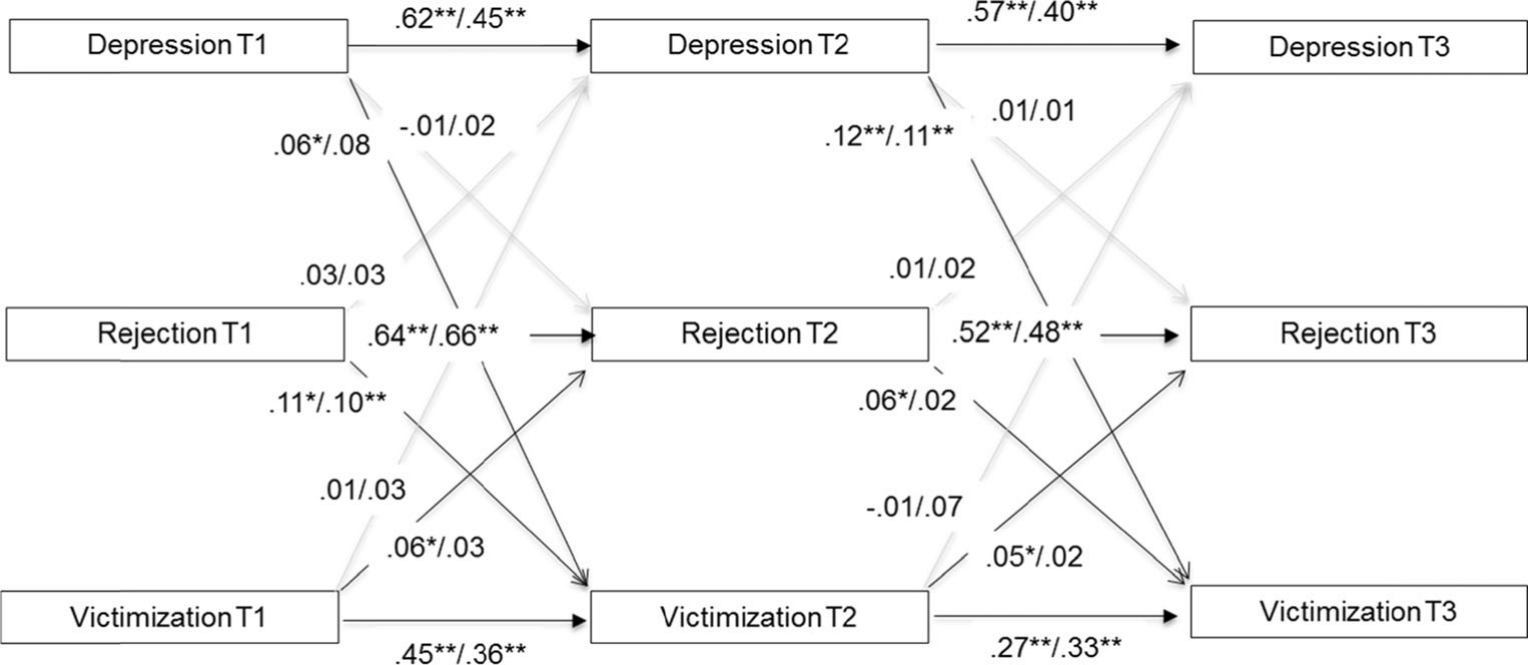
T1 = Time 1, T2 = Time 2, T3 = Time 3. Standardized associations between depression, peer rejection, and victimization before the dash for girls (*n* = 2871) and behind the dash for boys (*n* = 2774). Concurrent associations are controlled but not shown here. * = *p* < 0.05, ** = *p* < 0.01

**Fig. 2:**
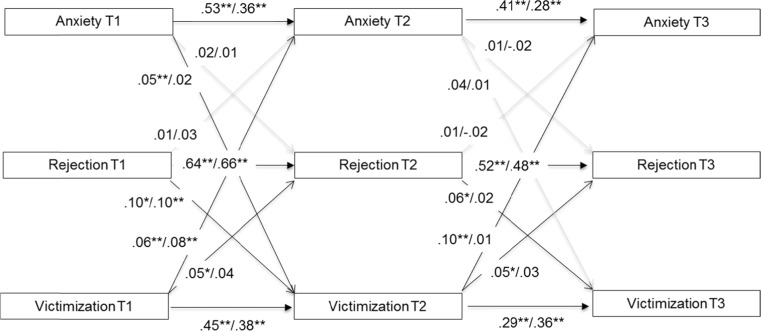
T1 = Time 1, T2 = Time 2, T3 = Time 3. Standardized associations between anxiety, peer rejection, and victimization before the dash for girls (*n* = 2871) and behind the dash for boys (*n* = 2774). Concurrent associations are controlled but not shown here. * = *p* < 0.05, ** = *p* < 0.01

Satorra-Bentler comparisons of model fit revealed significant gender differences in cross-lagged associations between depression, rejection, and victimization (TRd = 155.16) as well as in those between anxiety, rejection, and victimization (TRd = 95.76) based on *∆df* = 30 and *χ* ^*2*^ critical value =43.77. The two models were thus computed and interpreted separately for boys and girls. Both gender-variant models showed excellent model fit (for both: RMSEA =0.000, 95 % CI [0.000,0.017]; CFI = 1.00; SRMR = 0.006). The standardized estimates for each path as shown in Figs. 1 and 2 correspond to effect size estimates.

#### Depression

Figure 1 depicts the model with longitudinal associations between depression, peer rejection, and victimization. Concurrent associations between depression and victimization were substantial across all three time points for both genders (not depicted in the figure; β ranged from 0.24–0.42, *p* < 0.01). Moreover, after correcting for stability of the constructs and concurrent associations, depression was consistently predictive of victimization over time but not vice versa, providing empirical evidence for a symptoms-driven model. More specifically, for girls depression at T1 and T2 predicted victimization at T2 and T3, respectively, and for boys this association was found only from T2 to T3. Concurrent associations between peer rejection and depression were found only at T1 (both genders; β = .14, *p* < 0.01) and T2 (boys only; β = 0.06, *p* < .05) but no longitudinal associations existed. Lastly, reciprocal associations between victimization and peer rejection were consistently found for girls, but not for boys. For girls, the model explained 39–52 % of the variance in depression at T2 and T3 as well as 21–26 % of the variance in victimization and 43–47 % of the variance in peer rejection (all *p* < 0.01). For boys, these percentages were somewhat lower but still substantial, with 23–27 % explained variance in depression at T2 and T3, 19–20 % explained variance in victimization, and 45–50 % of explained variance in peer rejection (all *p* < 0.01).

#### Anxiety

The model with longitudinal associations between anxiety, peer rejection, and victimization is depicted in Fig. 2. While correcting for stability in all constructs, victimization consistently predicted anxiety for girls across time, and for boys the prediction from victimization to anxiety existed only between T1 and T2. In addition, for girls the opposite relation was also found, that is, anxiety at T1 predicted victimization at T2. Thus, for girls but not for boys we found reciprocal associations between anxiety and victimization over time. These findings suggest that for girls, a transactional model applies whereas for boys, the finding are in line with an interpersonal risk model. Concurrently, anxiety was associated with victimization at T1 (both genders; β = 0.25, *p* < 0.01) and at T2 (boys only; β = 0.10, *p* < 0.05). No longitudinal associations were found between peer rejection and anxiety, whereas concurrently this association was only found at T1 (β = 0.13, *p* < 0.01 for girls and β = 0.07, *p* < 0.01 for boys). Percentages of explained variance in anxiety at T2 and T3 differed considerably between boys (13–16 %) and girls (31–36 %). The model explained 43–47 % of the variance in peer rejection for girls and 45–50 % for boys, and 21–26 % of the variance in victimization for girls and 18–19 % for boys (all *p* < 0.01).

#### Indirect Effects

To follow up on the above two models for girls, which suggest indirect paths from internalizing problems to peer rejection via victimization, we included two additional paths to test whether the indirect effects were statistically significant. For depression, the paths from depression to victimization (β = 0.06, *p* < 0.05), and from victimization to peer rejection (β = 0.05, *p* < 0.05) were significant (see also Fig. 1), but the direct path from depression T1 to peer rejection T3 was not (β = 0.02, *p* = 0.54) and neither was the indirect effect (β = 0.003, 95 % CI [0.000, 0.005], *p* = 0.10). Similar paths were tested for the anxiety model in girls, and again the paths from anxiety to victimization (β = 0.06, *p* < 0.01), and from victimization to peer rejection (β = 0.05, *p* < 0.05) were significant (see also Fig. 2), as was the direct path from anxiety T1 to peer rejection T3 (β = 0.05, *p* < 0.05). However, the indirect effect of anxiety, via victimization, on peer rejection was not significant (β = 0.002, 95 % CI [0.000, 0.005], *p* = 0.08). Thus, peer victimization did not act as a mediator between depression or anxiety and peer rejection.

## Discussion

In this study we investigated the direction of associations between victimization, peer rejection, and internalizing problems in a sample of adolescents after they transitioned to secondary school. We tested three competing theoretical perspectives within one comprehensive model and further differentiated between anxiety and depression, and between genders. The three theoretical models guided our study and the interpretation of previous findings, but given the inconsistency in these findings together with methodological challenges and different age groups that were included, we had no specific hypotheses on differences between the depression and anxiety models regarding direction of effects. We did hypothesize to find stronger associations between all study variables (irrespective of the direction of the effects) for adolescent girls as compared to adolescent boys, given girls’ heightened vulnerability for depressive symptoms and importance of social relations (e.g., Angold et al. 2002; Stice et al. 2004). Overall, the current study showed that associations with victimization were different for depression and anxiety and that the models differed between genders.

First, in line with a symptoms-driven model we found that depression is predictive of subsequent victimization for both genders, although for girls this association was consistently found over the course of a year whereas for boys this only held in the first half of the school year. Thus, in our cross-lagged model the other direction (victimization leading to depressive symptoms) did not hold. This seems to contradict some previous studies among children (Arseneault et al. 2006; Williford et al. 2012), but in these studies the direction from depression to victimization was not included in the analytical model. However, our findings do concur with other studies that examined cross-lagged path models of depression and victimization (Tran et al. 2012; Vaillancourt et al. 2013). Our findings support the claim that depressive symptoms might be associated with social impairments, such as a decreased sociability, increased hostility, and difficulties in negotiating conflicts (Rudolph et al. 1994) which may cause peer difficulties including victimization. However, depression in itself might also elicit victimization given the submissive and withdrawn behavior that is typically part of a depressive state. Thus, the mechanism through which depression leads to subsequent peer difficulties in adolescence is an important direction for future research (see Agoston and Rudolph 2013).

Second, other theoretical models applied to our findings regarding anxiety for which clear gender differences were found. For girls, in line with a transactional model, it was found that anxiety was related to subsequent victimization and, simultaneously, victimization was related to subsequent anxiety. More than for depression, inconsistencies in associations with victimization were previously reported for anxiety and studies that focused on internalizing problems did often not differentiate between depression and anxiety (e.g., Vaillancourt et al. 2013) or focused on depression only (e.g., Kochel et al. 2012). Our findings on transactional paths between anxiety and victimization for girls are in line with Lester et al. (2012) although they reported no gender differences. For boys, we only found statistically significant paths between victimization and subsequent anxiety which corresponds to the interpersonal risk model. Thus, anxiety does not seem to put boys at risk for peer victimization whereas for girls our results point to a vicious cycle in which victimization and anxiety feed off each other.

It might be that the extent to which (and the ways in which) anxiety is manifested in behaviors differ between adolescent boys and girls. Given the differences between boys and girls with respect to the endeavors in social relationships (e.g., Rose 2002; Stice et al. 2004), maybe (social) anxiety in girls is more likely to interfere with their social behaviors and relationships which makes them vulnerable for victimization. Another, related, explanation may be found in the type of victimization. For example, some research suggests that relational victimization (e.g., spreading rumors, exclusion) is related to depression and anxiety among girls, and that physical victimization is related depression and anxiety in boys (Vuijk et al. 2007). This finding concurs with the assumption that girls place more importance on social relationships, and boys more on status and visibility. In our study we did not differentiate between types of victimization, which may explain the different associations with anxiety for boys and girls. However, this is only speculation and more research is needed to fully understand these gender differences. Taken together, our findings highlight the necessity to differentiate between anxiety and depression and between genders to understand the associations with peer relations.

Interestingly, neither depression nor anxiety was longitudinally related to peer rejection. Although some concurrent associations were found, after accounting for stability and longitudinal associations with peer victimization, depression and anxiety were no direct consequence or antecedent of peer rejection. Previous studies found that depressed adolescents are more often left alone (e.g., Kaltiala-Heino et al. 2010) but these studies did not control simultaneously for stability or paths in opposite direction. Another study found that depression was related to subsequent peer rejection via social helplessness (Agoston and Rudolph 2013), but social helplessness and peer rejection were measured via teacher reports while peer victimization was not included, which may have led to different associations. Instead, our findings suggest that among girls, both anxiety and depression are only indirectly related to peer rejection (cf. Kochel et al. 2012) although the indirect effect via victimization was not statistically significant. Peer rejection was however consistently related to victimization in both directions among girls. It can be assumed that rejected adolescents are easy targets for bullies because associating with a rejected classmate might damage someone’s own position in the peer group (e.g., Sentse et al. 2013). Bullies may thus fear less retaliation by their classmates when they target rejected peers. Being victimized (further) decreases one’s status in the group, which may lead to a vicious cycle of peer rejection and victimization. Importantly, this only applied to girls and not to boys, which confirms our hypotheses on the heightened importance and associated risks of (negative) social relations for adolescent girls.

Another explanation for why neither depression nor anxiety was longitudinally related to peer rejection might be found in the developmental stage (i.e., early adolescence) of our study participants. We argued that adolescence is an important age period to study associations between peer relations and psychopathology, because peer relations become increasingly important while (1) adolescents’ social status in the classroom needs to be (re)established due to the transition to secondary school and (2) most depressive and anxiety symptoms have their onset in early adolescence and increase and peak in adolescence (Hankin and Abramson 2001; Twenge and Nolen-Hoeksema 2002). However, the latter might also imply that dealing with depression and social anxiety, including displaying behaviors associated with these emotional difficulties, is normative especially in adolescence. As such, it may not necessarily stem from or lead to peer rejection. In contrast, at a younger age it might be less normative to deal with depression and anxiety which would therefore stand out more to the peer group and may eventually lead to peer rejection. In line with this, Kochel et al. (2012) found internalizing problems predictive of lower peer acceptance in late childhood (grades 4–6) whereas in the current study, no such associations were found in adolescence (grades 7–9). Future research should focus in more detail on these possible age-related differences in associations between peer relations and psychopathology.

### Strengths and Limitations

Strengths of the current study include that we formally tested three competing theoretical perspectives using advanced analytical methods that included all possible concurrent and longitudinal relations among the variables simultaneously. Second, peer rejection, victimization, and different forms of internalizing problems were collected at multiple time points and were reported by different informants. Finally, associations were tested among a large cohort and were separately conducted for depression and anxiety, and for boys and girls.

In reviewing the results, however, some shortcomings must be considered as well. First, small effect sizes were found. When using large cohorts, even small effects are likely to produce significant results. Replication of associations is needed in other samples in order to evaluate the theoretical significance of findings. We need to bear in mind though, that the relatively small effects are still highly significant considering that they were found above and beyond the strong stability paths, and together they led to relatively high explained variances in our variables. This means that despite small effect sizes, the practical significance of the results is substantial given that our results point to important pathways through which negative peer relations and psychopathology might develop.

Second, although we made use of different reporters, some associations may still have been inflated by shared method variance as both internalizing problems and victimization were self-reported. This may have caused same-reporter bias as well, as depression is related to cognitive distortions (Rudolph and Clark 2001) which may lead to a more negative view on peer relations and over-reporting of victimization. However, we believe that both internalizing problems and victimization, especially the more covert forms, are not easily noticeable for classmates and as such we used self-reported scales. Peer rejection was peer-reported and was still consistently associated with self-reported victimization. Still, future studies might do well to include multiple reporters, such as classmates, teachers or even parents.

Last, the time span in our study is relatively short. Although we had three measurement points, the total time span between them was one year and it might be that the underlying processes we studied need more time to unfold. This might also be the reason why the indirect (mediated) paths from internalizing problems to peer rejection were not significant. To draw any conclusions regarding these indirect effects, more studies are needed that cover a greater span in time.

## Conclusion

Taken together, this study was one of the first attempts to longitudinally examine the direction of associations between internalizing problems, social status, and peer victimization in one model and as such contributed to the extant knowledge on these associations among adolescents. Knowledge about the antecedents and consequences of victimization is important for preventing bullying in classrooms. Although most effective anti-bullying programs are school-, classroom-, or group based (see Ttofi and Farrington 2011) extra attention should be paid to depressed and anxious adolescents, especially in secondary school when support from peers is of increasing importance but at the same time more difficult to obtain. One example could be that within an anti-bullying program, students do not only learn to recognize bullying and how they can step in to help, but also how to recognize and deal with students suffering from internalizing problems. As such, adolescents may become more understanding and more aware of anxious and depressed feelings which may help prevent these students of becoming victimized or rejected. Further research is necessary to clarify *why* depressed and anxious adolescents are more vulnerable to be victimized by classmates, particularly by examining the role of personal factors (such as cognitions, self-regulation, social skills, and withdrawal) and group/classroom factors (e.g., social climate, teacher characteristics, group levels of bullying and victimization). Understanding these mechanisms would further contribute to the prevention of peer difficulties and lower the risks of internalizing problems.
